# Atomoxetine: toxicological aspects of a new treatment for attention deficit hyperactivity disorder in Brazil

**DOI:** 10.47626/2237-6089-2024-0798

**Published:** 2025-09-18

**Authors:** Gabriel Christian de Farias Morais, Shopnil Akash, Edilson Dantas da Silva Junior, Claudio Bruno Silva de Oliveira, João Firmino Rodrigues-Neto, Umberto Laino Fulco, Shahina Akter, Jonas Ivan Nobre Oliveira

**Affiliations:** 1 Departamento de Biofísica e Farmacologia Universidade Federal do Rio Grande do Norte Natal RN Brazil Departamento de Biofísica e Farmacologia, Universidade Federal do Rio Grande do Norte (UFRN), Natal, RN, Brazil.; 2 Department of Pharmacy Daffodil International University Dhaka Bangladesh Department of Pharmacy, Daffodil International University, Sukrabad, Dhaka, Bangladesh.; 3 Hospital Pediátrico Maria Alice Fernandes Natal RN Brazil Hospital Pediátrico Maria Alice Fernandes, Natal, RN, Brazil.; 4 Escola Multicampi de Ciências Médicas UFRN Caicó RN Brazil Escola Multicampi de Ciências Médicas, UFRN, Caicó, RN, Brazil.; 5 Bangladesh Council of Scientific & Industrial Research Dhaka Bangladesh Bangladesh Council of Scientific & Industrial Research, Dhaka, Bangladesh.

## Abstract

**Objective:**

Atomoxetine is widely used in the treatment of attention deficit hyperactivity disorder (ADHD), offering reduced risks of adverse motor effects and chemical dependence. However, its pharmacokinetic properties and toxicological risks require further exploration. This study aimed to predict the physicochemical profile, medicinal chemistry characteristics, and ADMET (absorption, distribution, metabolism, excretion, and toxicity) properties of atomoxetine using *in silico* web-based tools.

**Methods:**

Physicochemical, medicinal chemistry, and pharmacokinetic parameters of atomoxetine were analyzed using predictive computational models. Emphasis was placed on properties that influence drug efficacy and safety, particularly in the context of ADHD treatment.

**Results:**

*In silico* analyses revealed that atomoxetine may carry potential risks of hepatotoxicity, cardiotoxicity, neurotoxicity, nephrotoxicity, respiratory system toxicity, skin toxicity, and carcinogenicity. These predicted toxicological effects highlight the importance of further investigation into atomoxetine’s safety, especially across diverse patient populations and varying durations of treatment.

**Conclusion:**

The findings from this predictive study suggest that careful monitoring of atomoxetine use is warranted in clinical settings. Furthermore, additional controlled studies are needed to develop personalized dosing protocols that account for individual variability in metabolism and toxicity response, enabling a safer and more effective use of the drug.

## Introduction

Attention deficit hyperactivity disorder (ADHD) is a prevalent neuropsychiatric condition frequently identified in the pediatric and adolescent populations. It manifests across a spectrum, influencing aspects of learning, emotional responses, cognitive processes, and social behaviors, to varying extents,^1,2^ and is commonly associated with other mental disorders and/or substance use.^3^ The symptoms of ADHD fall into three main areas: inattention, hyperactivity, and impulsivity.^4^ Neurochemically, ADHD is linked to dysregulations in signaling within various brain regions, with a notable emphasis on the prefrontal cortex (PFC),^5^ and is directly related to catecholamines, dopamine, and noradrenaline.^6^ Atomoxetine is employed in the management of ADHD across diverse age groups, ranging from children to adults.

The exact mechanism of action of atomoxetine remains unclear. However, it is believed to be associated with its selective inhibition of presynaptic norepinephrine reuptake in the PFC. Atomoxetine exhibits a high affinity and selectivity for norepinephrine transporters, while demonstrating minimal to no affinity for either dopamine or serotonin transporters or various other neurotransmitter receptors.^7,8^ Moreover, it has been reported that atomoxetine modulates cortical synaptic dopamine uptake via the nonspecific action of noradrenaline transporters in the PFC, selectively increasing dopamine levels in this area without affecting dopamine levels in the motor or reward-related areas of the striatum. This mechanism ameliorates the symptoms of ADHD without causing the same motor side effects or abuse liability as other stimulants.

In Brazil, the advent of atomoxetine signifies a milestone in ADHD treatment. Historically dominated by stimulants such as methylphenidate and lisdexamfetamine, the therapeutic landscape has been transformed by the introduction of atomoxetine, offering a more comprehensive approach.^9^ Particularly tailored for ADHD patients with concomitant conditions like tics, anxiety, sleep disorders, and substance use disorder, atomoxetine emerges as a therapeutic option with notable advantages.^10^

A pivotal attribute of atomoxetine lies in its antidepressant action, conferring benefits for individuals grappling with the depressive and anxious symptoms that often co-occur with ADHD.^11^ In stark contrast to stimulants, atomoxetine exhibits a diminished propensity for inducing substance abuse or misuse. The selective binding of atomoxetine to noradrenergic transporters, without affecting dopamine neurotransmission in the PFC, minimizes the risks associated with psychoactive substance abuse and misuse. This innovative therapeutic approach offers a promising alternative for ADHD management, significantly enhancing the overall well-being of affected individuals.^12^

However, it has been reported that atomoxetine may trigger or exacerbate psychotic or manic symptoms in children and adolescents with ADHD. Although these effects are rare, cases of hallucinations, delusions, mania, or agitation have been reported in patients without a history of psychotic or manic illness who were given atomoxetine at normal doses. These symptoms can be very distressing and require immediate intervention. It is therefore recommended that patients starting treatment with atomoxetine be carefully monitored, especially those with risk factors for psychotic or bipolar disorders, such as family history of psychosis, substance abuse, or trauma.^13^

Understanding pharmacokinetics is crucial for determination of the mechanism of action, therapeutic effects, and potential adverse effects of drugs.^14^ Although the pharmacokinetics of atomoxetine are well understood, additional studies are crucial to further extend understanding its pharmacokinetics. Such studies can provide additional insights into the drug’s absorption, distribution, metabolism, and elimination, optimizing dosing regimens and enhancing the safety and efficacy of atomoxetine.^15^ Another important aspect is to investigate the toxicological potential of atomoxetine, as it has only recently become available in Brazil and there is still little data on its long-term safety and efficacy.^16^ In fact, information on the toxicity of atomoxetine is considered scarce and is primarily derived from case studies involving overdose.^17^

*In silico* pharmacokinetic studies constitute potent methodologies employing computational techniques to predict the pharmacokinetic attributes Absorption, Distribution, Metabolism, Excretion (ADME) and Toxicity (ADMET) of drugs, based on their molecular structures. Such investigations facilitate efficient and cost-effective drug discovery, playing an indispensable role in drug research, development, and refinement by providing early insights into the behavior of potential drug candidates within the body. They aid researchers in prioritizing compounds with favorable properties by anticipating parameters such as bioavailability, permeability, metabolism, and potential adverse effects, thereby guiding decision-making in drug design and optimization. These computational approaches serve as invaluable instruments for refining drug dosing, recognizing potential safety concerns, and ultimately enhancing the efficiency and success rate of the drug development trajectory.^18,19^ Therefore, this study aimed to further assess the physicochemical profile and medicinal chemistry characteristics of atomoxetine, alongside its pharmacokinetic properties – specifically, absorption, distribution, metabolism, and excretion – as well as its potential toxicology (ADMET) through use of web-based *in silico* tools. This was conducted to obtain insights that may aid in the planning of future studies focused on evaluating the therapeutic regimen, efficacy, and safety of atomoxetine.

## Methods

We employed an *in silico* method to evaluate the pharmacokinetic and toxicity profile of atomoxetine. This method utilizes computerized models capable of predicting the drug’s physicochemical and medicinal chemistry characteristics as well as the processes of absorption, distribution, metabolism, excretion, and toxicity of the drug within the human body. This approach reduces the cost, time, and use of animals in toxicology testing and provides relevant information for drug development and clinical use.^20^

Initially, the Simplified Molecular Input Line Entry Specification (SMILES) notation of atomoxetine was obtained from PubChem (https://pubchem.ncbi.nlm.nih.gov), an open-access chemistry database. Next, the canonical SMILES for atomoxetine (CC1=CC=CC=C1OC(CCNC)C2=CC=CC=C2) was input to various web-based *in silico* pharmacokinetics tools to predict several parameters related to physicochemical and ADMET properties. These tools included DrugBank (https://go.drugbank.com/), pkCSM (https://biosig.lab.uq.edu.au/pkcsm/), admetSAR (http://lmmd.ecust.edu.cn/admetsar2), PreADMET (https://preadmet.webservice.bmdrc.org/), ADMETlab 3.0 (https://admetlab3.scbdd.com/), and PRED-HERG (http://predherg.labmol.com.br/) (accessed November 2023).

These well-validated platforms were chosen because they offer comprehensive ADMET prediction due to their improved accuracy and performance, utilization of large datasets, advanced machine learning algorithms, user-friendly interfaces, and broad coverage of diverse ADMET endpoints.

### Ethical considerations

This study did not involve human participants, animals, or sensitive personal data, and therefore did not require approval from an ethical review board. The work is purely computational in nature. All data used in the experiments were obtained from publicly available datasets and do not contain identifiable information or raise ethical concerns.

## Results

**Figure 1:**
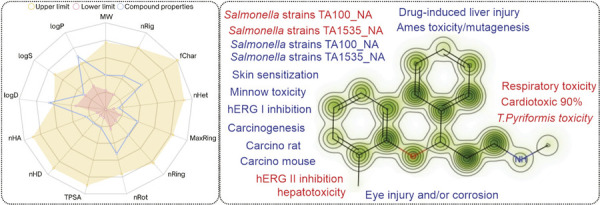
Physicochemical descriptors and toxicity-related properties of atomoxetine from DrugBank, pkCSM, admetSAR, PreADMET, ADMETlab 2.0, and PRED-HERG. fChar = formal charge; logD = logP at physiological pH; logP = log of the octanol/water partition coefficient; logS = log of the aqueous solubility; MaxRing = number of atoms in the biggest ring; MW = molecular weight; nHA = number of hydrogen bond acceptors; nHD = number of hydrogen bond donors; Het = number of heteroatoms; nRig = number of rigid bonds; nRing = number of rings; nRot = number of rotatable bonds; TPSA = topological polar surface area.

The physicochemical descriptors for atomoxetine were as follows, molecular weight (MW): 255.16 g/mol; number of hydrogen bond acceptors (nHA): 2.0; number of hydrogen bond donors (nHD): 1.0; number of rotatable bonds (nRot): 6.0; number of rings (nRing): 1.0; number of atoms in the largest ring (MaxRing): 6.0; number of heteroatoms (nHet): 2.0; formal charge (fChar): 0.0; number of rigid bonds (nRig):12.0; topological polar surface area (TPSA): 21.26; log of the aqueous solubility (logS): -3.16 log mol/L; log of the octanol/water partition coefficient (logP): 3.41 log mol/L; and logP at physiological pH 7.4 (logD): 2.936 log mol/L. All descriptor values were within the recommended limits for a molecule with pharmacological properties, with the exception of logP, which had a value greater than the upper limit (predicted logP for atomoxetine was 3.41 log mol/L, while compounds in the range from 0 to 3 log mol/L are considered proper) (Figure 1).

Analyzing the physicochemical descriptors described above, we found that atomoxetine passed the Lipinski rule (MW ≤ 500; logP ≤ 5; Hacc ≤ 10; Hdon ≤ 5), the GlaxoSmithKline (GSK) rule (MW ≤ 400; logP ≤ 4), and the golden triangle rule (200 ≤ MW ≤ 500; -2 ≤ logD ≤ 5), indicating that this compound would have a favorable ADMET profile. However, atomoxetine was rejected according to the Pfizer rule (logP > 3; TPSA < 75), which suggested a potential for toxicity. The basic medicinal chemical descriptors showed favorable results for the measure of drug-likeness based on the concept of desirability (quantitative estimate of drug-likeness [QED]) of 0.85 (suitable value > 0.67), synthetic accessibility (SAscore: easy), synthetic accessibility score (GASA: easy), and natural product-likeness score (NPscore) of -0.06 (values in the range of -5 to 5 are considered suitable). However, the number of sp3 hybridized carbons/total carbon count (Fsp3) for atomoxetine was 0.29, which is lower than the suitable value of 0.42 and could be related to poor solubility.

The analysis of absorption predictors revealed favorable results for Caco-2 permeability, Madin-Darby canine kidney (MDCK) permeability, human intestinal absorption (HIA), and oral bioavailability (F50%), suggesting that this compound would have good oral absorption, permeability, and bioavailability. On the other hand, atomoxetine seems to have a high probability of being an inhibitor of P-gp. The prediction results for atomoxetine distribution showed a plasma protein binding fraction of 97.8% (optimal < 90%) and a fraction unbound in plasma (Fu) of 1.6% (optimal: > 5%), which could indicate a low therapeutic index for this drug. Optimal values for volume of distribution (VD) and blood-brain barrier permeability were predicted for atomoxetine. The *in silico* prediction of atomoxetine metabolism classified this compound as a substrate or inhibitor of different CYP450 isoforms as follows: substrate for CYP1A2, CYP2C19, CYP2C9, CYP2D6, CYP3A, and CYP2B6; inhibitor for CYP1A2, CYP2D6, and CYP3A4. Regarding excretion, a moderate clearance of 8.96 mL/min/kg and a short half-life of 1.182 h were predicted for atomoxetine.

**Figure 2:**
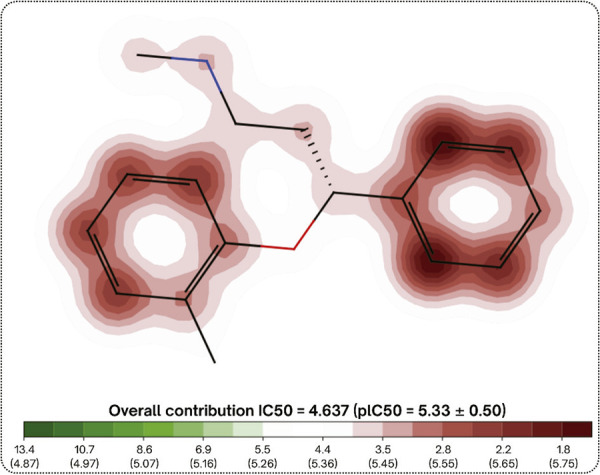
Aryloxy group of atomoxetine (highlighted in red) obtained using the ADMETlab 2.0 tool. IC = inhibitory concentration.

**Figure 3:**
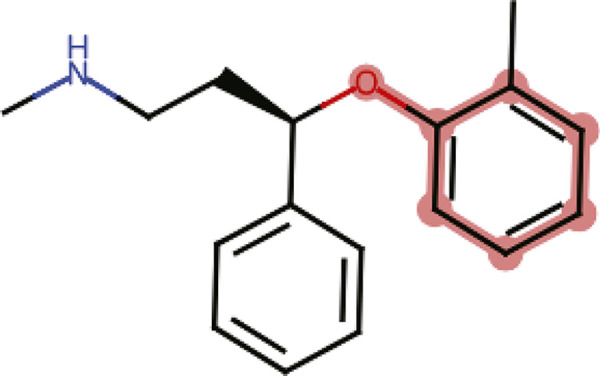
The figure shows atomoxetine analyzed for cardiac toxicity using the PRED-HERG tool. The inhibitory concentration (IC)50 indicates the concentration of the substance that inhibits 50% of the activity of the hERG channel. The lower the IC50, the greater the risk of cardiac toxicity. The red areas in the image indicate the toxic potential of the chemical groups or structures that contribute to blocking the hERG channel. The IC50 value shown is the sum of the contributions of the individual groups or structures, the plC50 value is the negative logarithm used to compare the potency of the substance.

Atomoxetine has an aryloxy group in its structure (Figure 2) and exhibits a potential for undesirable thiol reactivity when compared to a collection of more than 3,500 compounds. This could indicate that atomoxetine has the potential to interact with off-target proteins, inducing adverse/toxic effects.^21,22^ For the analysis of atomoxetine toxicity, an output value for each endpoint was predicted. The output value represents the probability of being toxic, ranging from 0 to 1. Atomoxetine presents a high prediction probability (range 0.8-1.0) of being an hERG blocker (inhibitory concentration [IC]50 ≤ 10 µM) (0.926) (Figure 3), and for inducing skin sensitization (0.929), respiratory toxicity (0.914), human hepatotoxicity (0.888), drug-induced nephrotoxicity (0.846), and drug-induced neurotoxicity (0.823). Moreover, an important predictive model for *Tetrahymena pyriformis* toxicity encompassing an extensive data set (1,571 chemicals) shows that atomoxetine has a high toxic potential for this microorganism, with a pIC50 of 1.26 log/ug/L. Atomoxetine also presented positive results for mutagenicity according to a predictive method for the Ames test, which use *Salmonella* strains TA100_NA and Salmonella strains TA1535_NA (Figure 1).

## Discussion

*In silico* pharmacokinetic and toxicology predictions for atomoxetine revealed concerns that should be carefully analyzed to obtain a personalized dosage and enhance the therapeutical safety of atomoxetine in clinical practice. First, it is important to highlight that the predicted number of sp3 carbon atoms (fsp3) could indicate that atomoxetine may have poor solubility, resulting in suboptimal ADME properties.^23,24^ Furthermore, according to the Pfizer rule, the predicted values of logP (3.41 log mol/L) and TPSA (21.26) for atomoxetine suggest that this drug is approximately 2.5 times more likely to be toxic than non-toxic.^25^

Prediction of the absorption properties of atomoxetine also revealed a high probability that this compound exerts inhibitory effects on P-gp. P-gp is an efflux transporter that mediates the ATP-dependent efflux of drugs from cells, leading to diminished intestinal permeation and limited bioavailability subsequent to oral administration.^26^ Thus, the inhibition of P-gp provoked by atomoxetine could result in increased systemic exposure to other drugs that are also substrates of P-gp, potentially altering their pharmacokinetics and efficacy and increasing the risk of toxicity. It was also predicted that atomoxetine may inhibit the activity of the P450 cytochrome isoforms CYP1A2, CYP2D6, and CYP3A4. However, *in vivo* studies have demonstrated that co-administration of atomoxetine with other drugs that are also substrates of CYP2D6 and CYP3A does not result in clinical implications.^27^

Chemically reactive compounds have the potential to modify off-target proteins, leading to adverse effects such as immunotoxicity and idiosyncratic hypersensitivity reactions.^21^ For these reasons, the assessment of intrinsic chemical reactivity of drug candidates is expected to provide important information for the early elimination of reactive compounds and accelerate the development of more selective drugs with fewer adverse effects *in vivo*. In this scenario, the aryloxy group present in the structure of atomoxetine could exhibit undesirable thiol reactivity, leading to significant toxicological consequences.^22^ The aryloxy group interaction with thiols results in formation of covalent adducts with protein thiol groups, which may cause nonspecific covalent interactions affecting many protein targets. This reactivity can be harmful as it initiates tissue damage through formation of thiyl radicals and “active oxygen” species, thereby inducing cytotoxic effects, hemolysis, and hepatotoxicity, among other harmful effects.^28,29^

Although atomoxetine is considered effective and generally well tolerated, there is evidence from the *in silico* toxicology predictions of possible organ and genome toxicities. Compared with the curated database, which contains 5,984 compounds with well-defined experimental end-points, atomoxetine poses a high risk for inhibition of hERG, which encodes the potassium channel involved in cardiac repolarization.^30^ Inhibition of hERG can lead to prolongation of the QT interval (an electrocardiographic parameter that indicates the duration of electrical systole – heart contraction), resulting in *torsades de pointes*, a potentially fatal ventricular tachyarrhythmia.^31^ Therefore, pre-clinical and clinical studies should be conducted to evaluate this predicted cardiotoxicity provoked by atomoxetine, which, if confirmed, may contraindicate its use in heart conditions or other situations that could prolong the QT interval, such as electrolyte disturbances, use of certain drugs (antidepressants, antipsychotics, and antiarrhythmics), and hypothyroidism.^32^

Furthermore, the *in silico* toxicology predictions yielded evidence of hepatotoxicity triggered by atomoxetine. This was revealed by the analysis of computational predictors of hepatotoxicity generated from a dataset comprising the chemical structure of 951 compounds reported to have a wide range of effects on the liver in different species, including humans, rodents, and non-rodents.^33^ Atomoxetine is mainly metabolized by the CYP2D6 isoform into 4-hydroxyatomoxetine (4-OH-ATX) and N-desmethylatomoxetine (N-DM-ATX),^34^ which can undergo further oxidation to form quinone imines. These are electrophilic species that can covalently bind to cellular macromolecules such as proteins and genetic material, generating oxidative stress. Glutathione and N-acetylcysteine are antioxidant agents that can bind to quinone imines and inactivate them, preventing cell damage. However, if glutathione and N-acetylcysteine levels are insufficient, quinone imines accumulate, causing hepatotoxicity. In their study of the CYP2D6-mediated metabolic activation of atomoxetine in rats,^35^ You et al. found glutathione and N-acetylcysteine conjugates of atomoxetine metabolites in liver microsome incubations. This indicates that these metabolites are potentially hepatotoxic and that atomoxetine can cause liver damage at high doses or in individuals with genetic polymorphisms affecting CYP2D6. As a result, polymorphisms associated with the cytochrome P450 genes that metabolize atomoxetine, mainly CYP2D6 and CYP2C19, alter its efficacy and the frequency of its adverse effects in the body.^36^

Atomoxetine also presented a high predicted probability of inducing skin sensitization, respiratory toxicity, nephrotoxicity, and neurotoxicity. However, this drug is not typically associated with direct skin, kidney, or respiratory system toxicity. Most of the known toxic effects of atomoxetine are derived from clinical cases of acute ingestion of supratherapeutic doses, which result in transient tachycardia, vomiting, and cognitive disturbances.^37^

A predictive model of the toxicity test using *T. pyriformis* showed that atomoxetine has a high toxic potential for this microorganism, indicating that this drug may have potential toxicity in humans or other organisms.^38^ More studies are needed to evaluate the toxic potential of atomoxetine, as well as post-marketing surveillance and risk-benefit analysis, especially considering long-term exposure to this drug.

It is accepted that among the various toxicological endpoints of chemical substances, mutagenicity and carcinogenicity are of great importance due to their serious effects on human health. Studies that systematically examined prescribed drugs have successfully identified compounds associated with cancer risk.^39,40^ In this context, the Ames test is a widely used method to test the mutagenic potential of a chemical compound against *Salmonella typhimurium* strains.^41^ The theoretical Ames test for atomoxetine suggested possible mutagenic activity for strains TA100NA and TA1535NA, although this fact alone does not make a drug a carcinogen. To our knowledge, based on current data, there is no available evidence to support or rule out any association between atomoxetine and cancer development. More research is needed to definitively identify any link between atomoxetine and carcinogenic potential.

## Conclusion

We believe that the predicted physicochemical parameters, medicinal chemistry properties, and ADMET endpoints reported here should be more closely monitored when atomoxetine is used in patients with ADHD. In addition, controlled studies describing reliable protocols for personalized dosing, taking into account multifactorial variability in metabolism efficiency and toxicologic potential, would enable a more robust assessment of the safety profile of atomoxetine.
